# Osteodifferentiated Mesenchymal Stem Cells from Bone Marrow and Adipose Tissue Express HLA-G and Display Immunomodulatory Properties in HLA-Mismatched Settings: Implications in Bone Repair Therapy

**DOI:** 10.1155/2014/230346

**Published:** 2014-04-30

**Authors:** Florent Montespan, Frédéric Deschaseaux, Luc Sensébé, Edgardo D. Carosella, Nathalie Rouas-Freiss

**Affiliations:** ^1^CEA, Institut des Maladies Emergentes et des Therapies Innovantes (IMETI), Service de Recherche en Hemato-Immunologie (SRHI), Hopital Saint-Louis, IUH, 1 avenue Claude Vellefaux, 75010 Paris, France; ^2^Universite Paris Diderot, Sorbonne Paris Cité, IUH, Hopital Saint-Louis, UMR_E5, IUH, 1, avenue Claude Vellefaux, 75010 Paris, France; ^3^Stromalab UMR UPS/CNRS 5273, EFS-Pyrénées-Méditerranée, U1031 Inserm, 31432 Toulouse, France

## Abstract

Mesenchymal stem cells (MSCs) are multipotent cells that can be obtained from several sources such as bone marrow and adipose tissue. Depending on the culture conditions, they can differentiate into osteoblasts, chondroblasts, adipocytes, or neurons. In this regard, they constitute promising candidates for cell-based therapy aimed at repairing damaged tissues. In addition, MSCs display immunomodulatory properties through the expression of soluble factors including HLA-G. We here analyse both immunogenicity and immunosuppressive capacity of MSCs derived from bone marrow and adipose tissue before and after osteodifferentiation. Results show that HLA-G expression is maintained after osteodifferentiation and can be boosted in inflammatory conditions mimicked by the addition of IFN-**γ** and TNF-**α**. Both MSCs and osteodifferentiated MSCs are hypoimmunogenic and exert immunomodulatory properties in HLA-mismatched settings as they suppress T cell alloproliferation in mixed lymphocyte reactions. Finally, addition of biomaterials that stimulate bone tissue formation did not modify MSC immune properties. As MSCs combine both abilities of osteoregeneration and immunomodulation, they may be considered as allogenic sources for the treatment of bone defects.

## 1. Introduction

Bone is among the most frequently transplanted tissues with about 1 million procedures annually in Europe. Despite their considerable disadvantages, including the risk of disease transfer and immunologic rejection, limited supply of bone, costs, and complications, allografts and autografts account for more than 80% of total graft volume. Significant growth opportunities exist for synthetic bone grafts in association with mesenchymal stem cells (MSCs) from autologous or allogenic sources as alternatives to biological bone grafts in orthopaedic and maxillofacial surgery [1, 2]. In a classical approach, bone tissue engineering consists of harvesting bone marrow from a patient, isolating MSCs by their adherence to tissue culture plastic, expanding and differentiating those cells in culture to a sufficient number, and then seeding them onto a suitable synthetic scaffold prior to implantation into the same patient [3].

MSCs can be isolated from different tissues including bone marrow (BM), adipose tissue (AT), and perinatal sources [4]. Many reports highlighted the immunomodulatory properties of MSCs relying on three main mechanisms: (1) cell cycle arrest of immune cells at the G1 phase, (2) direct interaction with immune cells, and (3) paracrine effect through secretion of various factors including HLA-G, prostaglandin E2, cytokines (TGF*β*, IL6, IL10, HGF, VEGF, etc.), and enzymes (indoleamine 2,3-dioxygenase and inducible nitric oxide synthase) [5–8]. Based on these tolerogenic properties, allogenic MSCs are currently tested in various clinical trials [9, 10].

HLA-G molecules expressed by mesenchymal stem cells fulfill an important function since blockade of HLA-G using HLA-G neutralizing antibodies could reverse MSC ability to (i) generate* in vitro* the expansion of CD4^+^CD25^+^ FoxP3^+^ regulatory T cells, (ii) inhibit the alloproliferative T cell response, and (iii) suppress the cytotoxic function of NK cells. These results show that HLA-G molecules, mainly soluble HLA-G5, actively contribute to the immunosuppressive properties exerted by MSCs [11, 12].

In clinical trials aimed at repairing bone defects, the main objective is to develop new biomaterials that simulate bone issue formation in combination with MSCs. In this context, our work entailed assessing, from an immunological perspective, whether allogenic MSCs could be used without a risk of rejection instead of autologous MSCs. The results obtained* in vitro* validate this hypothesis since the MSCs proved to be hypoimmunogenic and immunosuppressive in allogenic conditions. Moreover, following infusion in bone, MSCs may undergo osteodifferentiation process under the influence of* in vivo* osteogenic factors. We thus evaluated whether (1) allogenic MSCs committed to osteodifferentiation process can be rejected or not due to histoincompatibility and (2) combination with biomaterials modifies MSCs immune properties.

## 2. Materials and Methods

### 2.1. Isolation of PBMC

PBMC were isolated from blood of healthy volunteer donors (after informed consent) from the French Blood Establishment (EFS, Saint-Louis Hospital, Paris, France) by density-gradient centrifugation over Ficoll-Paque PLUS (GE Healthcare). These cells were used as HLA-mismatched responding cells in MLR.

### 2.2. *In Vitro* Osteodifferentiation

MSCs from BM or AT were obtained from Reborne consortium center (http://www.reborne.org/). Before experiments, MSCs were thawed and expanded through seeding 1000 cells/cm^2^ in T75 flasks. When cultures reach 60–70% confluence cells were harvested and seeded for immunological assays. The osteodifferentiated MSCs used as stimulating cells in MLR were obtained as previously described [13]. Briefly, MSCs were cultured in osteoblastic differentiation medium consisting of *α*MEM, FBS, ascorbic acid, NaH_2_PO_4_, and BMP-4 during 14 to 23 days.

### 2.3. Preparation of MSC-Biomaterial Complex For Immunological Tests

24-well (ultralow attachment) plates containing disks of MBCP+ (macroporous biphasic calcium phosphate) granules with the same diameter of each well bottom were prepared in collaboration with Biomatlante (France) to achieve a complete adherence of seeded MSCs to the biomaterial and then a complete induction of osteoblastic differentiation of MSCs. The disks of MBCP+ were washed for 48 hours before use to avoid any toxic effect on MSCs. Then MSCs were seeded on MBCP discs during 5 days in order to colonize homogenously the particles. Then, MSC-biomaterial complex was tested in mixed lymphocyte reactions.

### 2.4. Mixed Lymphocyte Reaction (MLR) Using MSC as Either Stimulating Cells Or Third-Party Cells Facing HLA-Mismatched PBMC as Responder Cells

PBMC from different donors were used as responder cells and MSCs or osteodifferentiated MSCs from BM or AT were used as either allogenic stimulating cells or third-party cells at various ratios. The HLA class II^+^ human B-lymphoblastoid cell line LCL 721.221 (ATCC) was used as positive control to stimulate HLA-mismatched PBMC. When LCL cells were used as stimulator cells, a 75 Gy dose irradiation was given and responder: stimulator ratio was of 1 : 0.5 with a final concentration of PBMC responder cells of 10^5^ cells/well in a 96-well flat bottomed plate.

Particularly, in MBCP experiments, MLR were performed in 24-well bottomed plates with a final concentration of PBMC responder cells of 10^6^ cells/well. Experiments comparing the effects of standard culture condition (no MBCP) and three-dimensional culture setting using MBCP discs were both performed in 24-well bottomed plates.

Cultures were incubated at 37°C in a humidified 5% CO_2_ air atmosphere. PBMC proliferation was measured at day 6 by [^3^H]-thymidine incorporation (1 *μ*Ci/well, Perkin Elmer) during the last 18 hours of culture. Cells were then harvested on filtermats A and thymidine incorporation into DNA was quantified, using a* beta* counter (Wallac 1450, Pharmacia). All samples were run in triplicate. The influence of MSC licensing with inflammatory cytokines such as IFN-**γ** at 10 ng/mL (Peprotech) and plus TNF-**α** at 15 ng/mL (R&D systems) was analyzed by adding these cytokines in cultures 48 hours before MLR [14].

### 2.5. Flow Cytometry

The cytokine (IFN-**γ** and TNF-**α**) treatment efficiency was checked by cytofluorometry analysis through the upregulation of HLA-DR expression on MSC. Briefly, cells were washed in PBS and stained with the anti-HLA-DR conjugated with PE (Beckman Coulter) in PBS 2% heat-inactivated fetal calf serum for 30 minutes at 4°C. Control aliquots were stained with an isotype-matched antibody to evaluate nonspecific binding to target cells. Fluorescence was detected by using the Epics XL4 flow cytometer (Beckman Coulter, Brea, CA, USA).

The expression of the HLA-G5 soluble isoform by MSCs was assessed by using the 2A12 mAb (Exbio) after cell permeabilization. The osteodifferentiation process was verified by cytofluorometry analysis through the induction of alkaline phosphatase (ALP) expression in osteodifferentiated MSC after cell permeabilization. Briefly, cells were first permeabilized by using saponin (Sigma) and then stained with 2A12 or anti-ALP (R&D systems) for 30 minutes at 4°C. After washing, cells were subsequently stained with an F(ab′)2 goat anti-mouse IgG antibody conjugated with PE (Beckman Coulter) for 30 minutes at 4°C. Control aliquots were stained with an isotype-matched antibody to evaluate nonspecific binding to target cells. Fluorescence was detected by using the Epics XL4 flow cytometer.

### 2.6. Statistical Analysis

Significance was assessed by using a nonparametric Mann-Whitney test, assuming a *P*  value < 0.05 as significant, and was marked with ∗ in the figures.

## 3. Results and Discussion

Two main questions were addressed in the present study relying on whether immune regulatory properties of MSCs are modified by (1) MSC differentiation towards the osteoblastic cell lineage or (2) the addition of synthetic biomaterial (i.e., MBCP+ granules). In this regard, we analyzed both the immunogenic and immunosuppressive properties of MSCs from BM or AT in allogenic conditions, that is, facing HLA-mismatched PBMC. To identify low immunogenic MSC types, we studied their ability to be recognized as allogenic cells by HLA-mismatched PBMC in MLR using MSCs as stimulating cells and PBMC from various healthy donors as responder cells. To examine their immunosuppressive properties, we studied their ability to modulate T cell alloproliferation as third-party cells in a classical MLR. All the functional experiments were performed by considering the differentiation status of MSC, either immature or osteodifferentiated, and seeded onto biomaterial or not.

**Figure 1:**
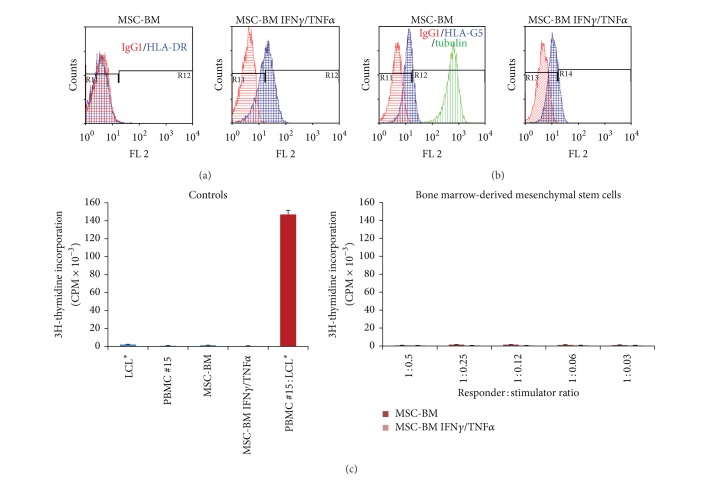
BM-derived MSCs express HLA-G and are hypoimmunogenic. (a) Expression of HLA-DR molecules was evaluated by flow cytometry analysis on BM-derived MSCs pretreated with IFN**γ** and TNF**α** (MSC-BM IFN**γ**/TNF**α**) or not (MSC-BM). IgG1 was used as isotype control Ab. (b) Expression of HLA-G5 was evaluated by intracellular flow cytometry analysis on BM-derived MSCs pretreated with IFN**γ** and TNF**α** (MSC-BM IFN**γ**/TNF**α**) or not (MSC-BM). Tubulin was used as positive control of cell permeabilization. (c) PBMC from healthy individual (#15) were used as responder cells towards BM-derived MSCs pretreated with IFN**γ** and TNF**α** (MSC-BM IFN**γ**/TNF**α**) or not (MSC-BM) as stimulating cells at various responder : stimulator ratios. Irradiated LCL* were used as positive control of T cell alloproliferation. Results are given as mean cpm ± s.e.m.; one representative experiment is shown.

**Figure 2:**
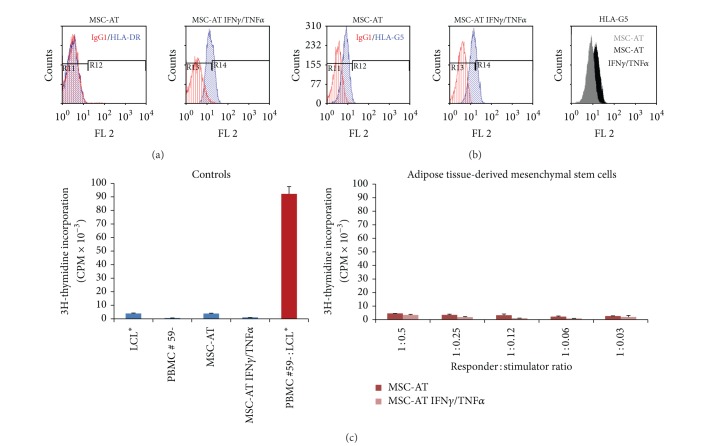
AT-derived MSCs express HLA-G and are hypoimmunogenic. (a) Expression of HLA-DR molecules was evaluated by flow cytometry analysis on AT-derived MSCs pretreated with IFN**γ**  and TNF**α** (MSC-AT IFN**γ**/TNF**α**) or not (MSC-AT). IgG1 was used as isotype control Ab. (b) Expression of HLA-G5 was evaluated by intracellular flow cytometry analysis on AT-derived MSCs pretreated with IFN*γ* and TNF**α** (MSC-AT IFN**γ**/TNF**α**) or not (MSC-AT). (c) PBMC from healthy individual (#59-) were used as responder cells towards AT-derived MSCs pretreated with IFN**γ**  and TNF**α** (MSC-AT IFN**γ**/TNF**α**) or not (MSC-AT) as stimulating cells at various responder : stimulator ratios. Irradiated LCL* were used as positive control of T cell alloproliferation. Results are given as mean cpm ± s.e.m.; one representative experiment is shown.

**Figure 3:**
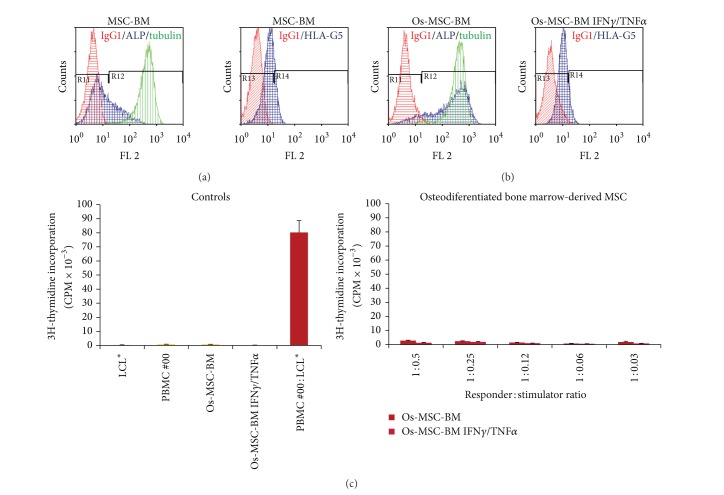
BM-derived MSCs committed to osteodifferentiation process express HLA-G and are hypoimmunogenic. (a and b) Expression of ALP and HLA-G5 was evaluated by intracellular flow cytometry analysis on BM-derived MSCs committed to 14-day osteodifferentiation process and pretreated with IFN**γ** and TNF**α** (Os-MSC-BM IFN**γ**/TNF**α**) or not (Os-MSC-BM). IgG1 was used as isotype control Ab. Tubulin was used as positive control of cell permeabilization. (c) PBMC from healthy individual (#00) were used as responder cells towards BM-derived MSCs committed to osteodifferentiation process and pretreated with IFN**γ** and TNF**α** (Os-MSC-BM IFN**γ**/TNF**α**) or not (Os-MSC-BM) as stimulating cells at various responder: stimulator ratios. Irradiated LCL* were used as positive control of T cell alloproliferation. Results are given as mean cpm ± s.e.m.; one representative experiment is shown.

**Figure 4:**
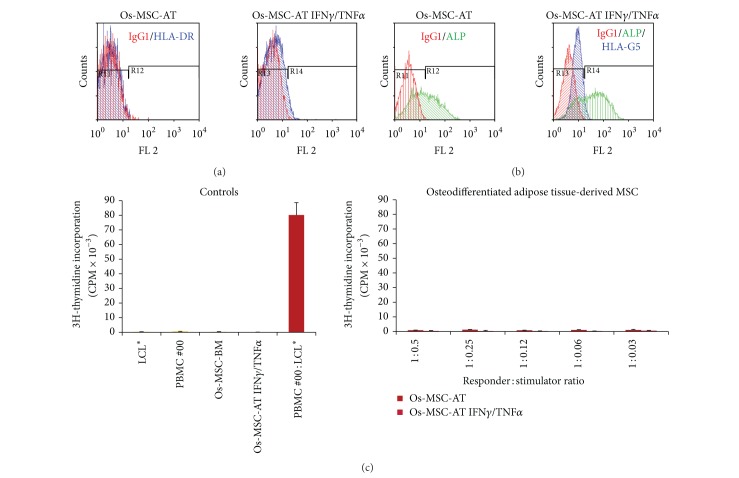
AT-derived MSCs committed to osteodifferentiation process express HLA-G and are hypoimmunogenic. (a) Expression of HLA-DR molecules was evaluated by flow cytometry analysis on AT-derived MSCs committed to 14-day osteodifferentiation process and pretreated with IFN**γ** and TNF**α** (Os-MSC-AT IFN**γ**/TNF**α**) or not (Os-MSC-AT). (b) Expression of ALP and HLA-G5 was evaluated by intracellular flow cytometry analysis on AT-derived MSCs committed to 14-day osteodifferentiation process and pretreated with IFN**γ** and TNF**α** (Os-MSC-AT IFN**γ**/TNF**α**) or not (Os-MSC-AT). IgG1 was used as isotype control Ab. (c) PBMC from healthy individual (#00) were used as responder cells towards AT-derived MSCs committed to osteodifferentiation process and pretreated with IFN**γ** and TNF**α** (Os-MSC-AT IFN**γ**/TNF**α**) or not (Os-MSC-AT) as stimulating cells at various responder : stimulator ratios. Irradiated LCL* were used as positive control of T cell alloproliferation. Results are given as mean cpm ± s.e.m.; one representative experiment is shown.

**Figure 5:**
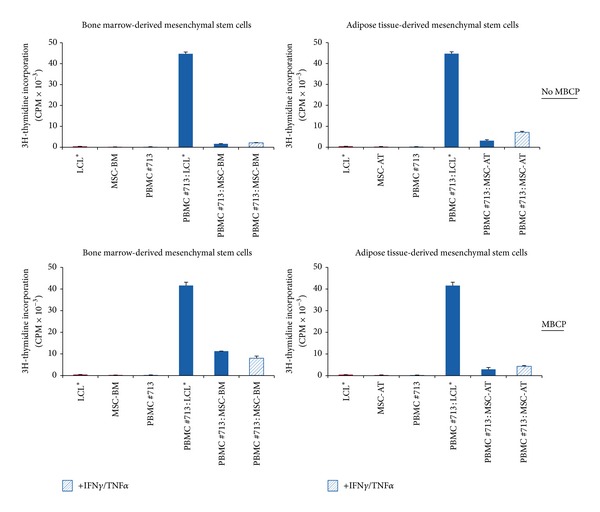
Both BM- andAT-derived MSCs when combined to MBCP biomaterial keep their hypoimmunogenicity. PBMC from healthy individual (#713) were used as responder cells towards either BM-derived MSCs (MSC-BM) or AT-derived MSCs (MSC-AT) that were pretreated with IFN**γ** and TNF**α** (IFN**γ**/TNF**α**) or not and used as stimulating cells at the responder : stimulator ratio of 1 : 0.2. MSCs were combined to MBCP biomaterial (MBCP) or not (No MBCP). Irradiated LCL* were used as positive control of T cell alloproliferation. Results are given as mean cpm ± s.e.m.; one representative experiment is shown.

No PBMC alloproliferation was observed in response to various doses of allogenic MSCs derived from bone marrow (Figure 1(c)) or adipose tissue (Figure 2(c)) even after licensing with IFN-**γ** and TNF-**α** (Figures 1(c) and 2(c)). Tables 1 and 2 summarize the results obtained with PBMC from distinct healthy donors. The efficiency of cytokine treatment was attested by the induction of HLA-DR expression on MSCs (Figures 1(a) and 2(a)). In order to evaluate the influence of osteodifferentiation process on the immunogenicity of MSCs, similar functional assays were performed using BM-derived and AT-derived MSCs committed to preosteoblastic MSCs as stimulating cells. The osteodifferentiation process was validated through the upregulation of ALP expression in osteodifferentiated MSCs (Figures 3(a), 3(b), 4(a), and 4(b)). Results show that both BM-derived and AT-derived MSCs committed to osteodifferentiation are still hypoimmunogenic whether they are pretreated or not with IFN-**γ** and TNF-**α** (Figures 3(c) and 4(c) and Tables 1 and 2). Then, we looked at whether combination of biomaterial (i.e., MBCP) with MSCs alters their immunogenicity. No differences were found between standard 2D-coculture conditions (MSC + PBMC) and 3D-coculture conditions (MSC + MBCP + PBMC). One representative allogenic combination is shown (Figure 5) for which the mean percentage of T cell alloproliferation is presented in Table 3 (*n* = 3 healthy donors). It is of note that the addition of MBCP to BM-derived MSCs treated or not with cytokines modifies slightly their immunogenicity, although no statistical difference was observed between both conditions (*P* > 0.1) (Table 3). Consequently, we can conclude that both the osteodifferentiation process and the presence of biomaterial (MBCP) did not abrogate the hypoimmunogenicity of MSCs.

Notably, expression of the tolerogenic soluble HLA-G5 protein was observed in MSCs derived from BM and AT and could be enhanced following treatment with IFN-**γ** and TNF-*α* (Figures 1(b) and 2(b)). Such enhanced expression of HLA-G by IFN-**γ** treatment was previously reported for other cell types such as monocytes [15], bronchial epithelial cells [16], thymic epithelial cells [17], and various tumor cells [18]. Nevertheless, HLA-G expression levels varied among the various batches of MSCs tested (data not shown). In agreement with recent findings, we observed enhanced HLA-G expression by MSCs following osteodifferentiation (Figures 3(a), 3(b), and 4(b)) [13].

**Figure 6:**
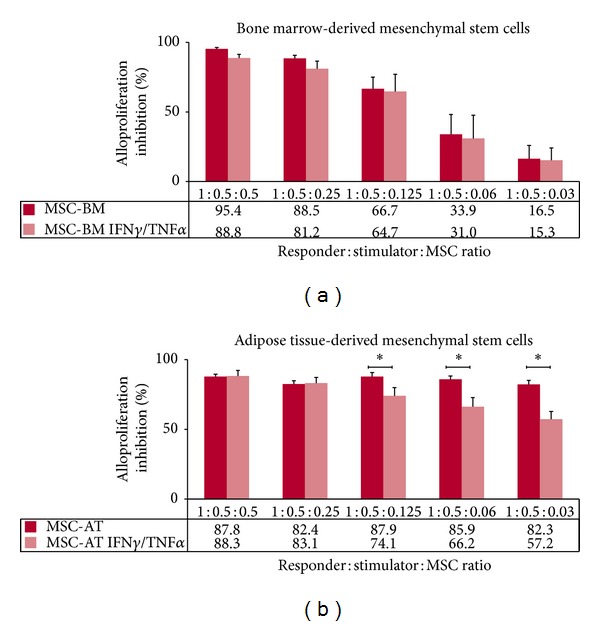
Both BM- andAT-derived MSCs display immunosuppressive properties in a dose-dependent manner. PBMC from healthy individuals were used as responder cells towards irradiated LCL* used as stimulating cells in presence of either (a) BM-derived MSCs (MSC-BM) or (b) AT-derived MSCs (MSC-AT) that were pretreated with IFN**γ** and TNF**α** (IFN**γ**/TNF**α**) or not and used as third-party cells at various responder : stimulator : MSC ratios. Results are given as mean percentage of alloproliferation inhibition ± s.e.m. according to the maximal alloproliferation observed with PBMC+LCL* using PBMC from 4 and 6 distinct healthy donors in BM- and AT-derived MSCs experiments, respectively.

**Figure 7:**
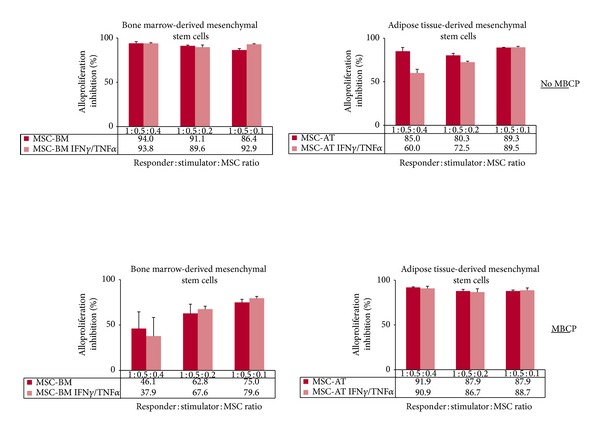
Both BM- andAT-derived MSCs when combined to MBCP biomaterial keep their immunosuppressive properties. PBMC from healthy individuals were used as responder cells towards irradiated LCL* used as stimulating cells in presence of either BM-derived MSCs (MSC-BM) or AT-derived MSCs (MSC-AT) that were pretreated with IFN**γ** and TNF**α** (IFN**γ**/TNF**α**) or not and used as third-party cells at various responder : stimulator : MSC ratios. MSCs were combined to MBCP biomaterial (MBCP) or not (No MBCP). Results are given as mean percentage of alloproliferation inhibition ± s.e.m. when compared to PBMC+LCL* using PBMC from 3 distinct healthy donors.

**Figure 8:**
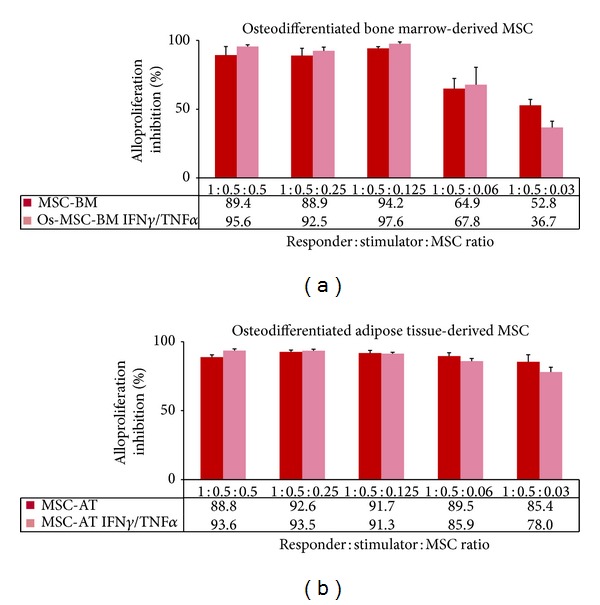
Both BM- andAT-derived MSCs when committed to osteodifferentiation process keep their immunosuppressive properties. PBMC from healthy individuals were used as responder cells towards irradiated LCL* used as stimulating cells in presence of either osteodifferentiated BM-derived MSCs (Os-MSC-BM) or AT-derived MSCs (Os-MSC-AT) that were pretreated with IFN**γ** and TNF**α** (IFN**γ**/TNF**α**) or not and used as third-party cells at various responder : stimulator : MSC ratios. Results are given as mean percentage of alloproliferation inhibition ± s.e.m. when compared to PBMC+LCL* using PBMC from 3 and 5 distinct healthy donors in BM- and AT-derived MSCs experiments, respectively.

Then, we investigated the immunomodulatory properties of MSCs as third-party cells in MLR. Results showed that both BM-derived and AT-derived MSCs display immunosuppressive properties in a dose-response manner (Figure 6). It is of note that (i) AT-derived MSCs are more potent at low doses compared to BM-derived MSCs (*P* < 0.05) (Figures 6(a) and 6(b)) and (ii) licensing with cytokines reduces significantly the immunosuppressive properties of AT-derived MSCs (*P* < 0.05) (Figure 6(b)). When combined to biomaterial (MBCP), both BM-derived and AT-derived MSCs still exert immunosuppressive properties as they greatly inhibit T cell alloproliferation with or without being seeded with MBCP (Figure 7). Although not statistically significant, addition of MBCP reduces the immunomodulatory properties of BM-derived MSCs at high responder: stimulator: MSC ratios. This could be due to steric hindrance when high numbers of cells are used. Indeed, such MBCP effect is no longer observed at low ratios (Figure 7). Also, both BM-derived and AT-derived MSCs when committed to preosteoblastic MSCs inhibit T cell alloproliferation and remain thus able to induce a tolerogenic microenvironment (Figure 8). Licensing with IFN-**γ** and TNF-**α** did not modify such MSC-derived immunosuppression (Figures 7 and 8). It is of note that once osteodifferentiated AT-derived MSCs are more potent at low doses compared to BM-derived MSCs as they display higher immunosuppressive effects (*P* < 0.05) (Figure 8). Such higher immunomodulatory capacity of adipose tissue-derived multipotent stromal cells compared to their bone marrow-derived counterparts has been previously reported [19].

Our present results are in agreement with previous reports showing that differentiation of stem cells does not alter their low immunogenicity and immunomodulatory properties. For instance, human amniotic epithelial cells, which have stem cell-like properties, retain their immunosuppressive functions after differentiation into hepatocyte-like cells [20]. Also, human Wharton's jelly-derived MSCs maintain the expression of immunomodulatory molecules, such as HLA-G, when subjected to osteogenic differentiation* in vitro* [21].

In conclusion, MSCs from BM or AT display tolerogenic properties which are maintained following osteodifferentiation process or addition of biomaterial and may thus be considered as allogenic sources for regenerating bone defects in orthopaedic and maxillofacial surgery [22].

## Figures and Tables

**Table 1:** MSC from BM after osteodifferentiation and licensing by IFN-*γ* and TNF-*α* do not induce PBMC proliferation in allogenic conditions.

R : S^a^ ratio	MSC-BM	MSC-BM IFN-*γ*/TNF-*α*	Os-MSC-BM	Os-MSC-BM IFN-*γ*/TNF-*α*
1 : 0.5	4.3 ± 2.1^b^	1.1 ± 0.3	3.3 ± 0.3	1.7 ± 0.2
1 : 0.25	4.5 ± 1.4	1.0 ± 0.3	3.1 ± 0.1	1.8 ± 0.5
1 : 0.12	5.3 ± 1.9	1.1 ± 0.4	3.2 ± 1.0	2.3 ± 1.2
1 : 0.06	5.4 ± 2.4	1.4 ± 0.5	2.6 ± 1.1	3.9 ± 3.1
1 : 0.03	4.3 ± 1.7	1.4 ± 0.5	2.2 ± 0.2	2.1 ± 1.2

^a^PBMC from healthy individuals were used as responder cells towards BM-derived MSCs pretreated with IFN-*γ* and TNF-*α* (MSC-BM IFN-*γ*/TNF-*α*) or not (MSC-BM) as stimulating cells at various responder : stimulator (R : S) ratios. Similar experiments were performed with BM-derived MSCs committed to osteodifferentiation process and pretreated with IFN-*γ* and TNF-*α* (Os-MSC-BM IFN-*γ*/TNF-*α*) or not (Os-MSC-BM) as stimulating cells.

^b^Data are mean ± s.e.m. of alloproliferation percentage obtained with 5 and 3 distinct healthy donors for MSC-BM and Os-MSC-BM experiments, respectively. This percentage is calculated considering PBMC stimulated with LCL* as 100% alloproliferation.

**Table 2:** MSC from AT after osteodifferentiation and licensing by IFN-*γ* and TNF-*α* do not induce PBMC proliferation in allogenic conditions.

R : S^a^ ratio	MSC-AT	MSC-AT IFN-*γ*/TNF-*α*	Os-MSC-AT	Os-MSC-AT IFN-*γ*/TNF-*α*
1 : 0.5	5.6 ± 1.0^b^	7.2 ± 3.1	1.1 ± 0.2	0.9 ± 0.6
1 : 0.25	5.5 ± 1.5	5.2 ± 2.6	1.3 ± 0.3	0.8 ± 0.5
1 : 0.12	5.3 ± 1.5	4.7 ± 2.9	1.0 ± 0.3	1.1 ± 0.8
1 : 0.06	5.3 ± 2.0	5.5 ± 3.0	1.0 ± 0.2	1.3 ± 1.0
1 : 0.03	4.6 ± 1.1	3.9 ± 2.0	1.4 ± 0.3	1.8 ± 1.3

^a^PBMC from healthy individuals were used as responder cells towards AT-derived MSCs pretreated with IFN-*γ* and TNF-*α* (MSC-AT IFN-*γ*/TNF-*α*) or not (MSC-AT) as stimulating cells at various responder : stimulator (R : S) ratios. Similar experiments were performed with AT-derived MSCs committed to osteodifferentiation process and pretreated with IFN-*γ* and TNF-*α* (Os-MSC-AT IFN-*γ*/TNF-*α*) or not (Os-MSC-AT) as stimulating cells.

^b^Data are mean ± s.e.m. of alloproliferation percentage obtained with 4 and 3 distinct healthy donors for MSC-AT and Os-MSC-AT experiments, respectively. This percentage is calculated considering PBMC stimulated with LCL* as 100% alloproliferation.

**Table 3:** Immunogenicity of MSC from bone marrow and adipose tissue combined with MBCP biomaterial and licensing by IFN-*γ* and TNF-*α*.

	MSC-BM	MSC-BM IFN-*γ*/TNF-*α*	MSC-AT	MSC-AT IFN-*γ*/TNF-*α*
No MBCP^a^	7.5 ± 2.2^b^	9.8 ± 4.0	13.9 ± 3.9	23.0 ± 5.2
MBCP	51.9 ± 14.1	41.1 ± 11.6	8.1 ± 0.8	11.0 ± 0.8

^a^PBMC from healthy individuals were used as responder cells towards BM- or AT-derived MSCs pretreated with IFN-*γ* and TNF-*α* or not as stimulating cells at 1 : 0.2 responder : stimulator ratio.

^b^Data are mean ± s.e.m. of alloproliferation percentage obtained with 3 distinct healthy donors. This percentage is calculated considering PBMC stimulated with LCL* as 100% alloproliferation.
